# Phenotypic integration and the evolution of signal repertoires: A case study of treefrog acoustic communication

**DOI:** 10.1002/ece3.3927

**Published:** 2018-02-22

**Authors:** Michael S. Reichert, Gerlinde Höbel

**Affiliations:** ^1^ School of Biological, Earth and Environmental Sciences University College Cork Cork Ireland; ^2^ Department of Biological Sciences University of Wisconsin‐Milwaukee Milwaukee WI USA

**Keywords:** anuran, complex signal, modularity, phenotypic integration, signal evolution

## Abstract

Animal signals are inherently complex phenotypes with many interacting parts combining to elicit responses from receivers. The pattern of interrelationships between signal components reflects the extent to which each component is expressed, and responds to selection, either in concert with or independently of others. Furthermore, many species have complex repertoires consisting of multiple signal types used in different contexts, and common morphological and physiological constraints may result in interrelationships extending across the multiple signals in species’ repertoires. The evolutionary significance of interrelationships between signal traits can be explored within the framework of phenotypic integration, which offers a suite of quantitative techniques to characterize complex phenotypes. In particular, these techniques allow for the assessment of modularity and integration, which describe, respectively, the extent to which sets of traits covary either independently or jointly. Although signal and repertoire complexity are thought to be major drivers of diversification and social evolution, few studies have explicitly measured the phenotypic integration of signals to investigate the evolution of diverse communication systems. We applied methods from phenotypic integration studies to quantify integration in the two primary vocalization types (advertisement and aggressive calls) in the treefrogs *Hyla versicolor*,* Hyla cinerea,* and *Dendropsophus ebraccatus*. We recorded male calls and calculated standardized phenotypic variance–covariance (**P**) matrices for characteristics within and across call types. We found significant integration across call types, but the strength of integration varied by species and corresponded with the acoustic similarity of the call types within each species. *H. versicolor* had the most modular advertisement and aggressive calls and the least acoustically similar call types. Additionally, **P** was robust to changing social competition levels in *H. versicolor*. Our findings suggest new directions in animal communication research in which the complex relationships among the traits of multiple signals are a key consideration for understanding signal evolution.

## 1 INTRODUCTION

Animal signals are composed of multiple components, and the relationship between these components determines the effectiveness of signals in eliciting responses from receivers (Bradbury & Vehrencamp, 2011). Understanding the significance of the interrelationships and covariances between trait components is the focus of the field of phenotypic integration (Klingenberg, 2010; Pigliucci, 2003; Pigliucci & Preston, 2004). Although phenotypic integration studies have largely focused on morphological traits (Cheverud, 1982; Goswami, 2006; Klingenberg & Zaklan, 2000), the philosophy and techniques of the field are also excellently suited to study the evolution of complex signal architectures. Like any complex phenotype, the strength of the interrelationships between the different components of signals determines variation in the expression of the signal as a whole, both within individuals as a result of plastic responses to environmental conditions (Plaistow & Collin, 2014; Schlichting, 1989) and between populations and species as a response to selection on correlated signal characteristics (Laughlin & Messier, 2015). The characteristics of tightly integrated signals will covary as a unit, whereas more modular subsets of signal components show strong integration within the subset but vary relatively independently from other subsets (Klingenberg, 2008; Murren, 2012). Phenotypic integration and modularity are key considerations for the study of the evolution of complex phenotypic traits, including signals, because the response to selection on one of a set of correlated characteristics depends on the strength and direction of selection acting on the other characteristics (Lande & Arnold, 1983; Phillips & Arnold, 1989; Schluter, 1996), potentially leading on the one hand to trade‐offs or expression of suboptimal phenotypes (Blows & Hoffmann, 2005; Kirkpatrick, 2009; Roff & Fairbairn, 2007) and on the other hand to rapid diversification in form when trait covariance facilitates the response to selection (Agrawal & Stinchcombe, 2009). Many recent studies have uncovered evidence for complex multivariate selection on signal form (Blows, Brooks, & Kraft, 2003; Brooks et al., 2005; Gerhardt & Brooks, 2009; Oh & Shaw, 2013; Tanner, Ward, Shaw, & Bee, 2017); it is thus important to also quantify the corresponding pattern of interrelationships among multiple component signal traits in order to understand how selection has shaped, and continues to act on, complex animal signals.

In addition to improving the understanding of the evolution of the different traits in a given animal signal, phenotypic integration techniques are also particularly well‐suited to the study of the evolution of the multiple signal types within a species’ repertoire. The signal repertoire consists of the set of different signal types, sometimes in multiple modalities (e.g., acoustic, visual, and chemical) and often used in different contexts, produced by individuals of a species (Bradbury & Vehrencamp, 2011). The evolution of complex signal repertoires has been hypothesized to have played a major role in diversification and the evolution of complex societies (Freeberg, Dunbar, & Ord, 2012; McComb & Semple, 2005; Pollard & Blumstein, 2012; Searcy, 1992), although support for these hypotheses remains tentative and little is known about how these multiple signals originated in the first place (e.g., Mason, Shultz, & Burns, 2014; Ord & Garcia‐Porta, 2012; Pitchers, Wolf, Tregenza, Hunt, & Dworkin, 2014). Phenotypic integration studies of signal repertoires may improve the understanding of the causes and consequences of the evolution of multiple signals because, as argued above for the traits of individual signals, measures of integration across signals quantify the potential constraints that act on the independent evolution of different signal types. The characteristics of different signals within a repertoire may be expected to covary positively to some extent if their production is controlled by a common morphological apparatus or physiological mechanism (Podos, 1997; Podos, Lahti, & Moseley, 2009), yet they may also be subject to conflicting selection pressures to optimize signaling in different contexts, for instance when certain magnitudes of signal characteristics are effective in one context but ineffective in another (Lane, Dickinson, Tregenza, & House, 2016; Leitão & Riebel, 2003; Moore & Moore, 1999) or if both signals draw from the same pool of energetic resources (Shutler, 2011). Nevertheless, while a growing number of studies are quantifying the complexity and interrelationships between components of animal (and plant; Junker et al., 2017) signals (Bertram, Fitzsimmons, McAuley, Rundle, & Gorelick, 2012; Blankers, Gray, & Matthias Hennig, 2017; Hebets et al., 2016; Moore, 1997; Pitchers et al., 2013), relatively little is known about the integration of characteristics across the signals in the repertoire (Wilkins, Shizuka, Joseph, Hubbard, & Safran, 2015).

The argument that phenotypic integration gives important insights into the response of traits to selection is based on the assumption that the phenotypic variance–covariance matrix (**P**), which is what is normally measured in phenotypic integration studies, is representative of the underlying genetic variance–covariance matrix (**G**), which determines the actual response to selection. There is much debate over whether **P** is a good predictor of **G** (Cheverud, 1988; Roff, 1996; Willis, Coyne, & Kirkpatrick, 1991). Estimates of the integration and modularity of behavioral characteristics such as signals, rather than morphological traits, may seem even less likely to correspond with underlying genetic relationships because of the highly plastic and context‐dependent nature of behavioral expression (Dochtermann, 2011). Nevertheless, there is reason to expect that tight phenotypic integration among some signal components reflects underlying genetic integration (see also Badyaev, 2004). First, multiple separate signal characteristics may trade off with one another because they share a common physiological or morphological constraint that limits the expression of certain combinations of signal characteristics. For instance, increasing either the rate or the duration of signals often entails increased energetic expenditure, leading to trade‐offs in the expression of these two traits (Reichert & Gerhardt, 2012; Wells & Taigen, 1986). Second, receivers evaluate multiple components of signals and often preferentially respond to specific ratios or combinations of signal characteristics, leading to correlated selection on the expression of multiple signal traits (Blows et al., 2003; Christensen, Mustaparta, & Hilderbrand, 1989; von Helversen, Balakrishnan, & von Helversen, 2004; Schul & Bush, 2002). If correlational selection is sufficiently consistent and strong, this can lead to genetic correlations, strengthening the correspondence between **P** and **G** (Lande & Arnold, 1983; McGlothlin, Parker, Nolan, & Ketterson, 2005; Sinervo & Svensson, 2002). Third, genetic architecture may directly constrain the variance in the expression of multiple sexual signal characteristics (Chenoweth & McGuigan, 2010; Walsh & Blows, 2009).

In this study, we examine the integration of two vocalization types used in mate advertisement and aggressive contexts in anuran amphibians. Anuran amphibians are tractable systems for the study of signal integration because their signals and signal repertoires are complex, but nonetheless generally limited compared to the extreme complexity seen in the signal repertoires of some other organisms (Marler, 2004; Sayigh, Quick, Hastie, & Tyack, 2013), and because the mechanistic underpinnings and evolutionary consequences of variation in signal structure are relatively well‐understood (Gerhardt & Huber, 2002; Wells, 2007). Most anuran signaling takes place in the context of reproduction, and acoustic signaling is the prominent modality in most species (Gerhardt & Huber, 2002). We studied the integration of acoustic signal characteristics in vocalizations produced in breeding aggregations by males of three species: the gray treefrog *Hyla versicolor*, the green treefrog *Hyla cinerea,* and the hourglass treefrog *Dendropsophus ebraccatus* (Hylidae). These species are similar in that all are prolonged‐breeding species that gather in choruses during the breeding season and produce both advertisement and aggressive calls. Advertisement calls are the primary call type for mate attraction, but also play a role in male–male competition (Wells, 2007). Aggressive calls are produced in the context of close‐range male–male interactions, and may play a role in assessment of intruding rivals (Bee, Reichert, & Tumulty, 2016), although they may also be involved in mate attraction in *D. ebraccatus* (Reichert, 2011). Advertisement and aggressive calls comprise the vast majority of vocalizations produced by these species; other call types such as release calls are produced extremely rarely and may indeed be variants of aggressive calls (Gerhardt, 2001; Pierce & Ralin, 1972). Furthermore, while vision plays a role in mate attraction and possibly aggression in these species (Laird, Clements, Hunter, & Taylor, 2016; Reichert & Höbel, 2015), it is not known whether visual signals, as opposed to cues, are involved, and acoustic signals are necessary and sufficient to elicit these behaviors (Gerhardt & Huber, 2002). Thus, the signal repertoire of these three species can be reasonably approximated by the two call types we studied.

**Figure 1:**
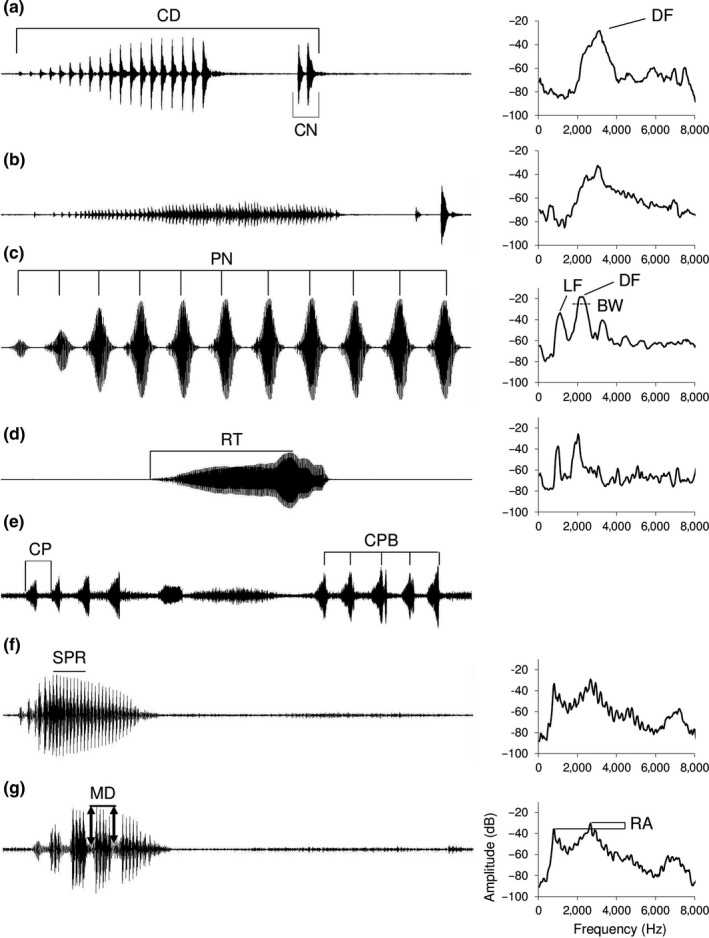
Waveforms (left) and power spectra (right) illustrating the advertisement and aggressive calls of the three study species and the measurement of different call characteristics (see also Table 1). (a) *Dendropsophus ebraccatus* advertisement call illustrating the measurement of call duration (CD), click note number (CN; CN = 1 in this example) and dominant frequency (DF). (b) *D. ebraccatus* aggressive call. (c) *Hyla versicolor* advertisement call illustrating the measurement of pulse number (PN; PN = 11 in this example), the low‐frequency peak (LF), and the interquartile bandwidth (BW). (d) *H. versicolor* aggressive call illustrating the measurement of rise time (RT). (e) A series of *H. versicolor* aggressive calls illustrating call period (CP) and number of calls per bout (CPB; CPB = 5 in this example). (f) *H. cinerea* advertisement call illustrating the region from which subpulse rate (SPR) was calculated. (g) *Hyla cinerea* aggressive call illustrating the calculation of modulation depth (MD) and relative amplitude (RA). The duty cycle (DC), bout duty cycle (BDC), and pulse rate (PR) are not illustrated because these are calculated from values of the characteristics illustrated here. Each waveform depicts a 500 ms section of a recording, except for (e), which depicts a 5‐s recording

Our first aim was to characterize integration and modularity in the signals and signal repertoires of each of the three study species and to make between‐species comparisons of the strength of integration. Integration refers to the strength of relationships across the different traits comprising a signal (or signal repertoire). Modularity refers to cases in which a subset of the traits making up a signal is strongly integrated, but with little covariance with other signal traits (in the case of signal repertoires, in which the traits making up one signal are strongly integrated with little covariance with traits in other signals). While integration and modularity are related concepts, each is best quantified and assessed with separate statistical techniques (see Methods). Our within‐species comparisons allow an assessment of the potential for independent expression and eventual evolution of different signal characteristics and identify constraints and trade‐offs acting on signal evolution. The between‐species comparisons indicate whether signal structure has indeed evolved divergently (Blows & Higgie, 2003). Such divergent evolution might be expected given between‐species variation in the acoustic similarity of advertisement and aggressive calls. The advertisement and aggressive calls of *D. ebraccatus* are qualitatively similar to one another, differing primarily in the pulse repetition rate (Figure 1a,b) (Reichert, 2013a; Wells & Schwartz, 1984), while those of *H. versicolor* are qualitatively different: Its advertisement calls consist of a long series of pulses with distinct pauses and its aggressive calls are much shorter and contain minimal amplitude modulation (Figure 1c,d) (Pierce & Ralin, 1972; Reichert, 2013b). The two call types of *H. cinerea* are intermediate in similarity: Only a small proportion of the advertisement call contains clear amplitude modulation, whereas the aggressive calls are clearly pulsed throughout (Figure 1f,g) (Gerhardt, 1978a). Thus, we predicted that *H. versicolor* would show the most modular and least integrated repertoire, while the repertoire of *D. ebraccatus* would be the most integrated. This discussion of acoustic similarity is based on obvious structural differences in the study species’ calls, but we note that similarity can be quantified for systems in which such classifications are less straightforward (Tchernichovski, Nottebohm, Ho, Pesaran, & Mitra, 2000).

Our second aim was to examine the stability of integration with changes in social competition. Plasticity integration concerns the extent to which **P** is stable across varying environmental conditions; in other words, whether plastic responses to environmental conditions are coordinated (i.e., integrated) across traits or whether each trait responds independently (Ellers & Liefting, 2015; Schlichting & Smith, 2002). The consequences of plasticity integration parallel those described above for phenotypic integration; in this case, there is the potential for trade‐offs or constraints on optimal signal expression within an individual across environments. Signal expression is generally highly dependent on environmental context, with signal characteristics varying with factors as diverse as temperature, habitat type, competition from conspecifics, and the presence of predators (Gerhardt, 1978b; Gross, Pasinelli, & Kunc, 2010; Patricelli, Krakauer, & Taff, 2016; Ziegler, Arim, & Narins, 2011; Zuk & Kolluru, 1998). However, while much is known about changes in individual signal characteristics in varying physical and social environmental conditions, it is unknown whether **P** itself is robust to such variation. If signal characteristics vary independently of one another, then the nature of multivariate signal evaluation (and, indeed, measurements of **P** by external observers) may be context‐dependent (Patricelli et al., 2016). Indeed, there is some evidence that for female evaluations of male‐mating signals, multivariate preferences are expressed differently under different environmental conditions (Reichert & Höbel, 2015; Reichert & Ronacher, 2015). In anurans, the level of competition in the social environment is a major driver of variation in calling behavior (Wells, 1988; Wells & Schwartz, 2007), and many species produce graded signals in which multiple song characteristics vary with the level of acoustic competition (reviewed by Bee et al., 2016). We tested whether these song characteristics in fact covary with one another across different levels of competition in the social environment, which would indicate that despite phenotypic plasticity, **P** itself is context‐independent. We tested the robustness of **P** to increased competition levels and variation in competitive status in *H. versicolor*, where data were available from interactions staged at different inter‐rival distances and where males are known to alter many individual components of their calls during intense vocal competition (Schwartz, Buchanan, & Gerhardt, 2002; Wells & Taigen, 1986).

We generated phenotypic correlation matrices of the call characteristics of each call type for each species and visualized the correlation matrix to depict the patterns of interrelationships between call characteristics. We then used matrix comparison techniques developed in studies of phenotypic integration to estimate the integration and modularity of the two primary acoustic signals in our study species’ repertoires and to test the effects of the social environment on signal integration. First, we tested whether, within each species, advertisement calls and aggressive calls are separate modules (i.e., with low covariation of signal characteristics between call types and high covariation within call types), and whether there is integration across call types (i.e., high covariation of signal characteristics across call types). Low modularity does not necessarily imply high integration, and separate hypothesis tests are necessary to evaluate levels of modularity and integration. Second, we tested whether the level of integration between advertisement and aggressive calls differed between the three species using a subset of call characteristics that were common to all species. This allowed us to test the hypothesis of a common **P** (i.e., common matrix structure) across species, which would imply limited divergence in signal architecture. Third, in *H. versicolor*, we tested whether **P** for advertisement calls is robust to increased levels of acoustic competition. We also tested whether **P** was robust to competitive status by comparing **P** between winners and losers of these vocal competitions. There are differences between the call characteristics of winners and losers (Reichert & Gerhardt, 2012, 2013a), but it is unknown whether there might be differences in the pattern of integration of winners’ and losers’ advertisement and aggressive calls. Together, these analyses illustrate a novel approach to the study of signal complexity and suggest hypotheses on the origins of diverse signal structures.

## 2 METHODS

### 2.1 Study species and recording techniques

The acoustic recordings of natural male calling behavior were obtained in the course of previous experiments, as described below for each species. In all cases, calls were recorded as 16‐bit WAV files (44.1 kHz sampling rate) with directional microphones (Sennheiser ME‐66, ME‐67, and ME‐80) onto digital audio recorders (Marantz PMD 660, PMD 661, PMD 670). Recordings of male *H. versicolor* were made as males interacted with one another during staged aggressive contests (*N *=* *167 contests involving 334 individuals) in a seminatural chorus within a greenhouse facility in Columbia, MO, USA (Reichert & Gerhardt, 2011). Males were captured from nearby ponds in Ashland, MO, USA, and placed within an artificial pond which resulted in nightly chorusing behavior. Subject males were initially positioned on wheeled platforms 1.8 m from one another, and we recorded at least 10 advertisement calls from each male before they were pulled to a distance of 0.9 m from one another. At this point, they were recorded for an additional 10 advertisement calls before the platforms were again pulled toward one another to the point that they abutted. At this point, males often gave aggressive calls in addition to advertisement calls; thus, our dataset includes recordings of advertisement and aggressive calls given by the same male within the same recording session. After the interaction, we noted the winner and loser (Reichert & Gerhardt, 2011) and measured each individual's body temperature with a cloacal thermometer. Recordings of *D. ebraccatus* were made from 16 pairs of males interacting with one another for 30 min in the field in Gamboa, Panama (see Reichert, 2011). During these interactions, most males produced both advertisement and aggressive calls. The average intermale distance was 3.2 m (Reichert, 2011). Male *H. cinerea* were recorded calling in response to either advertisement calls played back from a speaker placed 1 m from the focal male (*n *=* *20; average playback sound‐pressure level = 85 dB) or in response to an observer vocally mimicking a conspecific call (*n *=* *8). Males gave advertisement calls and a series of aggressive calls in response to both stimuli. In all cases, males were recorded at their natural calling perches at ponds at the East Texas Conservation Center in Jasper, TX, USA

### 2.2 Acoustic analyses

Call characteristics of advertisement and aggressive calls of each male were measured in Raven Pro 1.3 software (Cornell Laboratory of Ornithology). The specific characteristics measured depend on the species and call type because not all characteristics are present in all species’ signals (Table 1). However, a set of five characteristics was measured in all cases to facilitate comparisons between species (see Table 1 for definitions): (1) call duration, (2) dominant frequency (spectrogram settings: Hamming window, discrete Fourier transform size = 4,096 samples), (3) call period, (4) rise time, and (5) duty cycle. We measured additional characteristics in only certain species or call types to examine in more detail the patterns of integration within each species’ calls (Table 1). We note that some characteristics (duty cycle and pulse rate for *H. cinerea* aggressive calls) are derived entirely from other characteristics in the analyses. Duty cycle is the ratio of call duration and call period and therefore will naturally correlate with those variables. We nevertheless retained all three variables in the analyses because each is biologically relevant, because many of our analyses involve comparisons of these characteristics across the two different call types, and because different individuals tend to vary each of these three characteristics in different ways (Reichert & Gerhardt, 2012), implying potential differences in covariances that are of interest in this study. To determine whether including derived variables affected our conclusions, we repeated the measurements of integration and modularity excluding duty cycle for all species and aggressive call pulse rates for *H. cinerea* and present these results in Appendix 1.

**Table 1:** The call characteristics that were measured for each species and each call type. Frequency characteristics are indicated in bold

Characteristic	Abbr	Definition	Call type					
			Advertisement			Aggressive		
			*H.v*.	*H.c*.	*D.e*.	*H.v*.	*H.c*.	*D.e*.
Call duration	CD	Duration of the call, including any click note appendages in *D. ebraccatus*	x	x	x	x	x	x
**Dominant frequency**	**DF**	Frequency of maximum amplitude (Figure 1a)	x	x	x	x	x	x
Call period	CP	Amount of time from onset of one call to onset of the next	x	x	x	x	x	x
Rise time	RT	Amount of time from call onset to point of highest amplitude in call	x	x	x	x	x	x
Duty cycle	DC	Ratio of call duration and call period (estimate of acoustic “on time”)	x	x	x	x	x	x
Pulse number	PN	Number of pulses per call (in *H. cinerea* advertisement calls, the number of pulses in the pulsed portion of the call; Figure 1f)	x	x	x		x	x
Pulse rate	PR	Pulse number divided by call duration (in *H. cinerea* advertisement calls, the inverse of the average pulse period of the pulsed portion of the call)	x	x	x		x	x
Click notes	CN	Number of click note appendages (Figure 1a)			x			x
Calls per bout	CPB	Number of calls in a bout of aggressive calling (a sequence of calls in which no call period exceeded 1 s; Figure 1e)				x		
Bout duty cycle	BDC	Duty cycle calculated within a bout of aggressive calls				x		
Modulation depth	MD	Ratio between the maximum amplitude of the pulse of maximum amplitude within the call and the minimum amplitudes before and after that pulse (averaged over the pre‐ and postpulse ratios; Figure 1g)		x			x	
Subpulse rate	SPR	Rate at which subpulses were delivered within a pulse (Figure 1f; measured for 10 subpulses within advertisement calls or three within aggressive calls)		x			x	
**Relative amplitude**	**RA**	Amplitude difference between low‐frequency and high‐frequency peak (Figure 1g)		x			x	
**Low frequency**	**LF**	The peak value of the secondary, low‐frequency peak (Figure 1c)	x	x		x	x	
**Interquartile bandwidth**	**BW**	The difference in Hz between the frequency containing 25% of the energy in the call and the frequency containing 75% of the energy in the call (Figure 1c)	x		x	x		x

**Figure 2:**
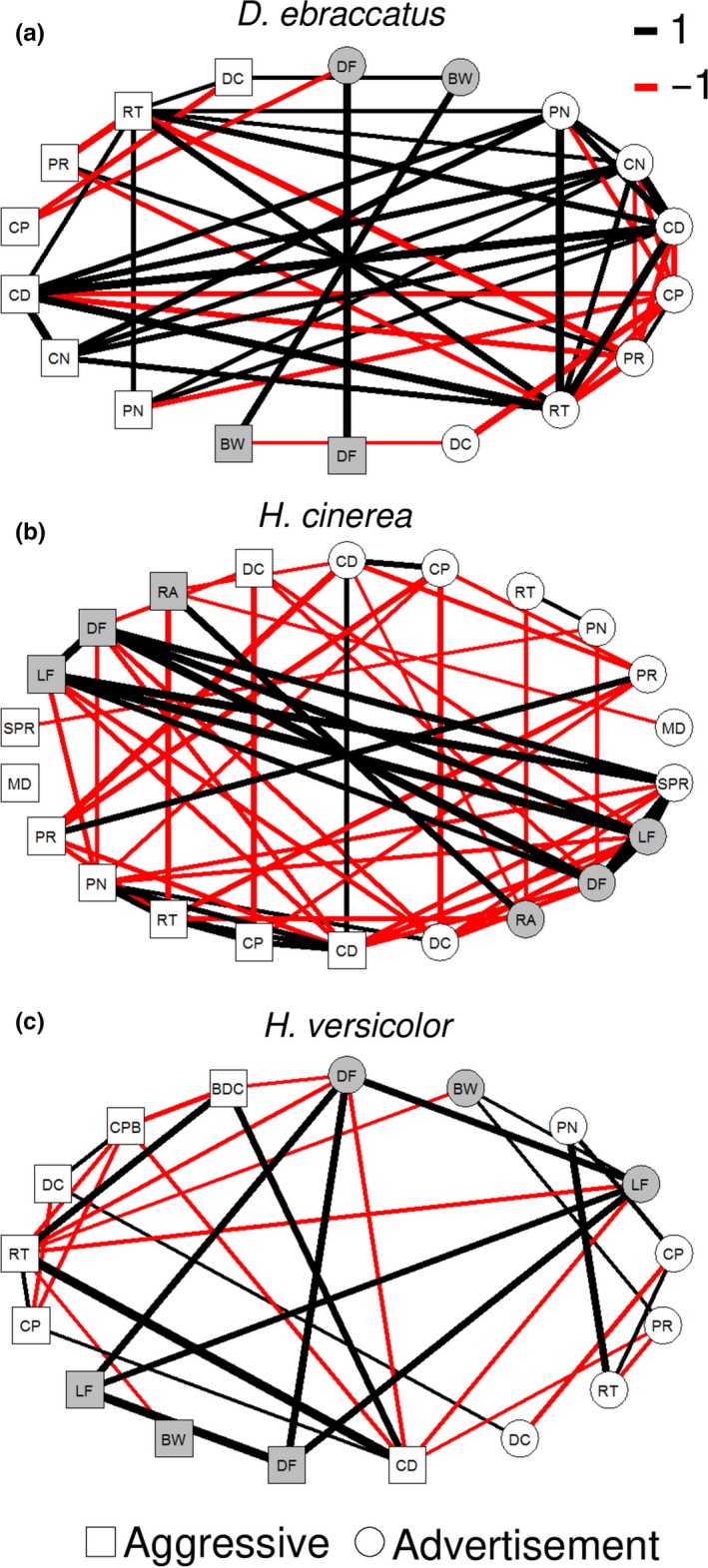
Correlation network for all call characteristics of advertisement (circles) and aggressive calls (squares) for the three study species. Line thickness indicates strength of correlation (value of Pearson correlation coefficient); positive correlations are denoted with black lines and negative correlations with red lines. Only robust correlations with a correlation coefficient greater than 0.3 are shown (see Methods). Abbreviations as in Figure 1

**Figure 3:**
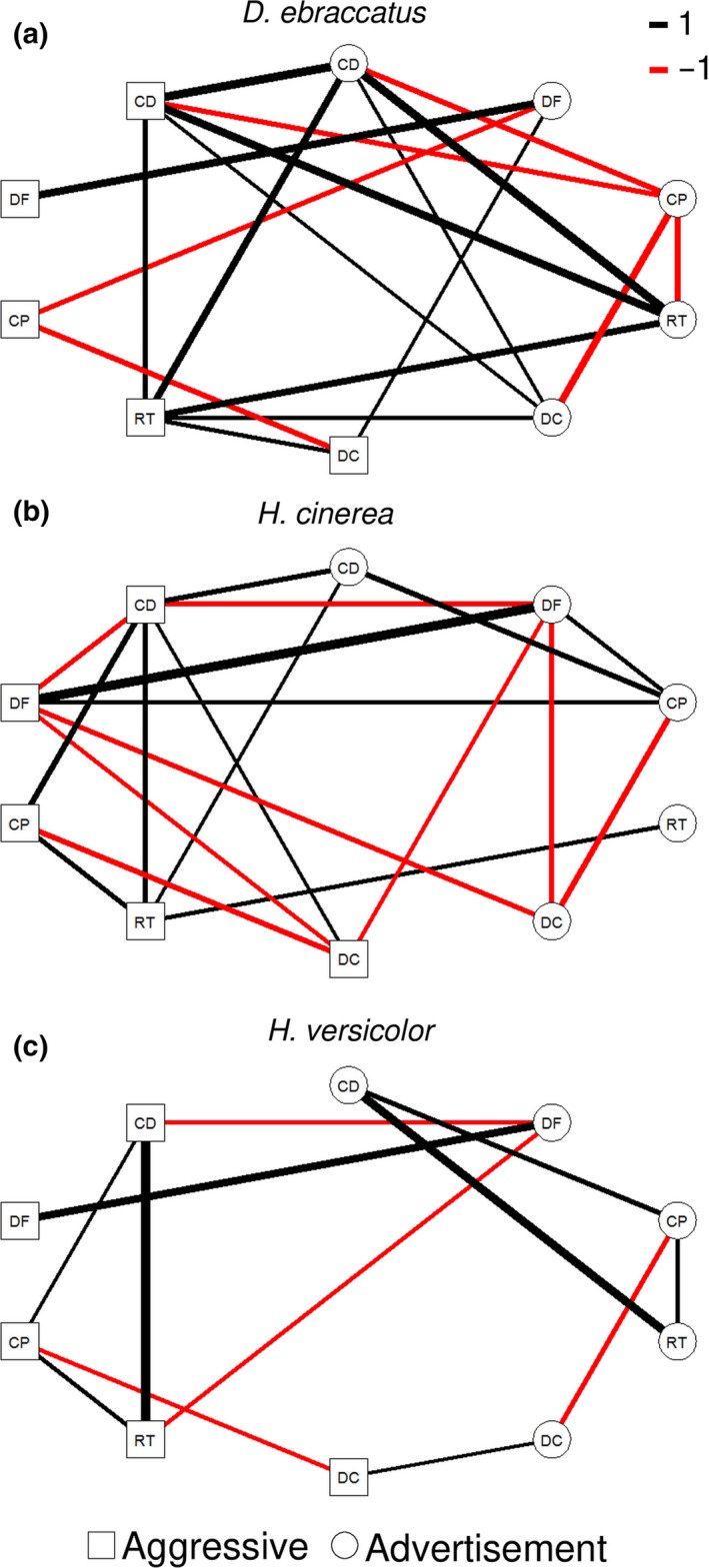
Correlation network for call characteristics common to both call types and all study species. Interpretation as in Figure 2. Abbreviations as in Figure 1

The second column gives abbreviations corresponding to those in Figures 2 and 3. An “x” indicates that the characteristic was measured for the given species and call type (*Hyla versicolor*:* H.v*.; *Hyla cinerea*:* H*.*c*.; *Dendropsophus ebraccatus*:* D*.*e*.). The first five characteristics listed are those common to all species and call types.

### 2.3 Data analysis

Only recordings for which we had measurements from at least five calls of a given call type from an individual were included in the dataset. We calculated mean values of each call characteristic of each call type for each individual. From this set of mean values, we then centered and scaled each call variable to a grand mean of zero and standard deviation of one. Thus, our analyses are based on correlation matrices rather than covariance matrices. We considered the use of correlation matrices more appropriate because the different call characteristics have very different absolute magnitudes and are measured on different scales. This data structure means that covariances would give potentially misleading indications of the relationships between variables (Goswami & Polly, 2010), which would be particularly troublesome for our comparisons between species with different absolute values of the measured call characteristics.

This procedure was performed separately for each call characteristic of each call type. In addition, for *H. versicolor*, in which advertisement calls were recorded from the same individuals at three different spatial positions, we calculated these values separately at each position. Unless otherwise noted, we used measurements from the position in which individual's platforms were abutting in analyses because the advertisement calls given at this position would have been in closest temporal proximity to the aggressive calls given by the same individuals (aggressive calls were only recorded at this position) and are thus the most relevant for joint analyses of the signal repertoire.

Sample sizes are given in the results tables associated with each test. Sample sizes varied because not all characteristics were measured for all individuals, and only individuals with a complete set of characteristics were included for a particular analysis. Thus, sample sizes depended on the analysis, for instance because some individuals produced only advertisement or only aggressive calls, or because temperature was not measured for some individuals. Finally, we note that individual data points for *H. versicolor* and *D. ebraccatus* are not strictly independent of one another because they were obtained from recordings of interacting individuals. We did not account for this possible dependence, but as our analyses are based on correlation matrices of many variables calculated across individuals, we consider this unlikely to have influenced our results.

Many, but not all, characteristics of frog calls vary with temperature (Wells, 2007), and therefore, temperature may affect the magnitude of correlations between call characteristics. Correcting for temperature, however, involves a linear transformation of data to that predicted at a common temperature and thus should have minimal to no effects on **P**. We therefore performed all analyses on call measurements that were not corrected for environmental temperature, but first confirmed that this was appropriate by comparing **P** before and after temperature correction. For *H. versicolor* and *H. cinerea*, calls were recorded at a wide range of temperatures (17.4–29.8°C, and 21.6–27.2°C, respectively). For *D. ebraccatus*, a tropical species, nightly temperature variation is minimal and temperatures were not recorded for the individuals examined in this manuscript. The following analyses therefore were only performed for *H. cinerea* and *H. versicolor*. We first used the parameters of linear regressions between temperature and each call characteristic to temperature‐corrected all call characteristics to a common temperature (chosen as the mean temperature of individuals with advertisement‐call recordings) of 23.4°C and 25.1°C for *H. versicolor* and *H. cinerea*, respectively. We then used the *z*‐scores and random skewers methods described below to compare the structure of temperature‐corrected and uncorrected **P** matrices containing (1) the combined set of advertisement and aggressive call characteristics, (2) advertisement‐call characteristics only, and (3) aggressive‐call characteristics only. These analyses were performed separately for each species’ mean call characteristics.

### 2.4 Visualization of signal integration

We visualized the correlation network structure by plotting the (Pearson's) correlations between different call characteristics using the methods of Wilkins et al. (2015). As these authors suggested, for the visualizations, we removed nonrobust correlation estimates by calculating 100000 bootstrapped correlation coefficients and discarding any resultant correlation between variables whose 95% confidence interval overlapped zero. We also do not depict any correlation coefficient lower than 0.3. However, we retained all variables in the statistical analyses below, which rely on comparing absolute values of correlation coefficients. Correlation magnitude and direction are denoted by the size and color of the lines connecting two call characteristics. All plots were created in the “qgraph” package (Epskamp, Cramer, Waldorp, Schmittmann, & Borsboom, 2012) for R version 3.2.2 software (R Development Core Team 2015).

### 2.5 Modularity and integration in advertisement and aggressive calls

In the analyses below, we perform separate statistical tests to evaluate the hypotheses that advertisement and aggressive calls are separate modules and that there is significant integration across the signal repertoire. Although modularity and integration are highly related concepts, a statistical test designed to test for a modular structure that fails to find evidence for modularity does not necessarily indicate significant integration and vice versa. Therefore, different statistical tests have been designed to best evaluate hypotheses of integration and modularity (Goswami & Polly, 2010; Klingenberg, 2008).

We tested the hypothesis that advertisement and aggressive calls act as separate modules; in other words that the covariance between characteristics within each call type is stronger than the covariance between characteristics across the two call types. We used the covariance ratio (CR; Adams, 2016) to assess the hypothesis of modularity. CR quantifies the covariation between hypothesized modules relative to the within‐module covariation and has a null value (indicating random covariation between variables) of 1. It is the direct comparison of covariation between modules relative to covariation within modules that makes the CR an appropriate test of modular structure, whereas other methods to describe covariance such as the partial least squares (PLS) analysis described below only quantify the covariation between groups of variables (Adams, 2016). CR coefficients significantly lower than 1 provide evidence for a modular structure. We calculated CR coefficients and assessed their significance using the modularity.test function in the geomorph version 3.0.4 package (Adams & Otárola‐Castillo, 2013) in R. For each species, we assigned its call characteristics into the hypothesized modules “aggressive call” and “advertisement call.” Statistical significance was evaluated by comparing the observed CR coefficient to the distribution of CR coefficients from 1,000 permutations in which the characteristics were assigned randomly to modules. *P*‐values were calculated as the proportion of permuted CR coefficients lower than the observed CR (Adams, 2016). Here, and elsewhere, we used an alpha of 0.05 to evaluate statistical significance. Separate statistical tests were performed for each species. Preliminary analyses suggested that frequency characteristics, which may strongly covary across call types because of a common relationship with body size (Gingras, Boeckle, Herbst, & Fitch, 2013), may have been driving the observed patterns of modularity. Thus, we performed these analyses both for all call characteristics and for only temporal call characteristics (i.e., those call characteristics associated with changes in the signal's amplitude over time).

We then tested the hypothesis that there is integration across the two call types. To test the hypothesis of integration, we used a PLS analysis implemented with the integration.test function in geomorph (Adams & Collyer, 2016). PLS estimates the axis of maximal covariation between two groups of variables (for details see Bookstein et al., 2003), and the statistical significance of PLS can be estimated by comparing the observed PLS coefficient to a distribution of values of coefficients obtained by random (*n* = 1,000 in our analyses) permutations across the two groups (Adams & Collyer, 2016). A large observed coefficient relative to this distribution is evidence for significant integration of characteristics. Note that, in contrast to the CR coefficient, the PLS analysis focuses only on the strength of covariation between groups of variables. For this reason, the hypotheses of integration and modularity are not mutually exclusive: It is possible to obtain evidence for both modularity and integration because the statistical methods used to test for each phenomenon examine different components of the same dataset. As above, separate statistical tests were performed for each species, and separate analyses were performed with all characteristics included and only temporal characteristics.

### 2.6 Common structure of **P** across species

We tested whether the pattern of integration across the call repertoire is similar in the three study species. For this analysis, we reduced our dataset to the five call characteristics common to each species and each call type (see Table 1), for a total of 10 call variables. We first calculated the level of integration within each species across the two different call types using PLS analysis as above. PLS coefficients cannot be directly compared as their value depends on sample size (Adams & Collyer, 2016). We therefore used the methods of Adams and Collyer (2016) to calculate *z*‐scores and associated confidence intervals for the PLS coefficients of the different species. We implemented this method using the compare.pls procedure in geomorph, which tests the null hypothesis of no difference in integration between species.

In addition, we compared the similarity of **P** calculated for each species’ common call characteristics using random skewers (Cheverud, 1996; Cheverud & Marroig, 2007). Random skewers is a method to assess matrix similarity that is particularly relevant for evolutionary studies because it quantifies the extent to which two matrices respond similarly to a common perturbation, which essentially simulates the response of a complex phenotype in two species to a common selection gradient (Lande, 1979). We multiplied each matrix by randomly generated selection vectors (1000 repetitions in which the elements of each vector were selected from a uniform distribution) and then calculated the vector correlations between the resulting vectors for each species. The magnitude of these correlations was compared to the magnitude of correlations calculated by applying the same method to a pair of covariance matrices that was randomly generated for each repetition (elements of these randomly generated matrices were also selected from a uniform distribution). We tested the null hypothesis of no common matrix structure using the skewers function (with the option “unifcorrmat” for the “covMethod” argument) in the phytools package (Revell, 2012) for R software.

Our analyses of integration and modularity included data from 28 *D*.* ebraccatus*, 28 *H*.* cinerea,* and 111 *H*.* versicolor* individuals. The method we used to compare integration between the species is designed to be sample‐size independent (Adams & Collyer, 2016). However, the statistical significance of the estimates of modularity and integration calculated from CR and PLS analyses, respectively, is dependent on sample size. Although we do not explicitly compare these measures between species, because the sample of *H. versicolor* individuals was much larger than that of the other two species, we investigated the sample‐size dependence of our results. To do this, we generated 1,000 samples of 28 individuals from the *H. versicolor* dataset (each replicate was sampled without replacement) and for each sample, we estimated modularity using CR, integration using PLS, and compared integration levels to those of the other species using the compare.pls procedure and random skewers. We present results from these analyses in Appendix 2.

### 2.7 Robustness of **P** to social environment

For *H. versicolor*, we had advertisement‐call recordings from males calling at three different intermale distances, representing escalating levels of acoustic competition. Trade‐offs between certain temporal characteristics emerge as acoustic competition increases (Reichert & Gerhardt, 2012), but it is unknown whether these effects apply to a broader set of call characteristics. We therefore used random skewers to compare the structure of **P** for advertisement‐call characteristics for male *H. versicolor* recorded at each of the three positions. We did not perform this analysis with aggressive‐call characteristics because aggressive calls were only recorded at the closest position.

The recordings for *H. versicolor* were made in the course of staged contests in which winners and losers could be identified (Reichert & Gerhardt, 2011). We used *z*‐scores and random skewers to compare the structure of **P** for winners and losers for (1) the combined set of advertisement‐ and aggressive‐call characteristics, (2) advertisement‐call characteristics only, and (3) aggressive‐call characteristics only.

## 3 RESULTS

Raw data for the individual call measurements are available from the Dryad Data Repository at https://doi.org/10.5061/dryad.d1k50. Raw **P** matrices for each analysis are presented in the Supporting Information. As expected, **P** changed only slightly following temperature correction in both *H. versicolor* and *H. cinerea* (Tables S1–S12). Comparisons of integration suggest similar levels of integration between advertisement and aggressive calls in temperature‐corrected and uncorrected formulations of **P** for each species (Table 2). Furthermore, in both species, random skewers analysis gave evidence for common matrix structure between temperature‐corrected and uncorrected measurements, both for advertisement and aggressive calls considered separately, and for a joint matrix containing both advertisement‐ and aggressive‐call characteristics (Table 2).

**Table 2:** Comparison of the strength of integration and the structure of **P** between temperature‐corrected and uncorrected values of call characteristics

Species	Call type	PLS comparison		Random skewers		*N*
		Comparison effect size	*p*	*r*	*p*	
*Hyla versicolor*	Adv	—	—	.98	<.001	202
	Agg	—	—	.998	<.001	157
	Adv and Agg	0.03	.49	.97	<.001	102
*Hyla cinerea*	Adv	—	—	.92	<.001	14
	Agg	—	—	.93	<.001	14
	Adv and Agg	0.34	.37	.9	<.001	13

The comparison of the strength of integration (PLS comparison) was performed only on **P** containing the full set of advertisement‐ and aggressive‐call characteristics for each species. The comparison of the structure of **P** (random skewers) was also performed separately for **P** containing either advertisement‐ or aggressive‐call characteristics only. For each pairwise species comparison, we give the effect size for the comparison of *r*_PLS_ values and associated *p*‐value (Adams & Collyer, 2016), and the correlation coefficient and *p*‐value from random skewers analyses. The null hypothesis for the PLS analysis is that there are similar levels of integration between corrected and uncorrected matrices; the null hypothesis for random skewers analysis is that there is no common structure between the corrected and uncorrected matrices.

### 3.1 Modularity and integration in advertisement and aggressive calls

For *H. versicolor*, when mean values of all call characteristics were included in the analysis, there was evidence that advertisement and aggressive calls are separate modules because the CR was significantly smaller than 1 (Table 3). Neither *D. ebraccatus* nor *H. cinerea* had CR coefficients that differed significantly from 1 (Table 3), and thus, there was no evidence for modularity of the call types in these species. This pattern may have been driven by the especially strong correlation between dominant frequencies of advertisement and aggressive calls. When we removed this characteristic so that the analyses only included temporal call characteristics, both *H. versicolor* and *D. ebraccatus* now showed evidence for a significant modular structure between advertisement and aggressive calls (Table 3). However, the CR coefficient for *D. ebraccatus* was very close to 1, indicating that modularity is at best weak (this result may also have depended on the inclusion of duty cycle as a variable; see Appendix 1). *H. cinerea* did not show evidence for modularity in either case (but see Appendix 1).

**Table 3:** Tests of modularity and integration of the signal repertoire

	Species	CR (CI)	*p*	*r*_PLS_	*p*	*N*
All characteristics	*D. ebraccatus*	1.03 (0.94–1.12)	.24	0.86	.001	28
	*H. cinerea*	1.06 (0.94–1.16)	.43	0.88	.001	28
	*H. versicolor*	0.76 (0.66–0.91)	.02	0.75	.001	111
Temporal characteristics only	*D. ebraccatus*	0.97 (0.88–1.08)	.048	0.85	.001	28
	*H. cinerea*	0.95 (0.80–1.15)	.12	0.75	.03	28
	*H. versicolor*	0.43 (0.35–0.64)	.001	0.41	.005	111

The CR statistic (with estimated 95% confidence interval) tests whether advertisement calls and aggressive calls are statistically separate modules. *r*_PLS_ tests whether there is significant integration across advertisement and aggressive calls. *N* indicates the number of individuals contributing mean values to the dataset.

Partial least squares analysis gave evidence for significant integration across advertisement‐ and aggressive‐call characteristics for all species, for both the full set of call characteristics and for only temporal characteristics (Table 3; Figure 2). However, the PLS coefficient was relatively low for *H. versicolor* temporal characteristics compared to the PLS coefficients for the other two species, and the statistical significance of the result for *H. versicolor* despite this relatively lower effect size was probably driven by the large sample size for this species (see Appendix 2). For this reason, to make direct comparisons between species in the level of integration of the signal repertoire, we needed to use a technique that is robust to variation in sample size, which we describe in the next section (Adams & Collyer, 2016).

### 3.2 Common structure of **P** across species

When comparing the structure of **P** only for those five characteristics that could be measured from each call type of each species (Tables S16–S18), there were no differences in the level of integration in pairwise comparisons between species (Table 4; Figure 3) using the *z*‐scores method of Adams and Collyer (2016). Furthermore, for comparisons between *H. versicolor* and both of the two other species, random skewers analysis gave evidence for commonalities in **P** between these species (i.e., some similarity in the pattern of integration across the call repertoire; Table 4). However, there was no evidence for a common **P** in the comparison between *D. ebraccatus* and *H. cinerea*, although *p *=* *0.08 (Table 4).

**Table 4:** Comparison of the strength of integration and the structure of **P** across advertisement and aggressive calls for the characteristics common to all species and call types

Metric	Species	PLS comparison		Random skewers	
		Comparison effect size	*p*	*r*	*p*
All characteristics	*H.v.–D.e*.	0.95	.17	.75	.001
	*H.v.–H.c*.	1.49	.07	.87	<.001
	*D.e.–H.c*.	0.42	.34	.67	.08
Temporal characteristics only	*H.v.–D.e*.	2.16	.015	.73	.049
	*H.v.–H.c*.	1.44	.074	.87	<.001
	*D.e.–H.c*.	2.90	.002	.69	.101

For each pairwise species comparison, we give the effect size for the comparison of *r*_PLS_ values and associated *p*‐value (Adams & Collyer, 2016), and the correlation and *p*‐value from random skewers analyses. Sample sizes for each species as in Table 3. The null hypothesis for the PLS analysis is that there are similar levels of integration between the two species; the null hypothesis for random skewers analysis is that there is no common matrix structure between the two species.

All species exhibited a strong correlation between the dominant frequency of advertisement and aggressive calls, which may have driven the finding of no differences in matrix structure. We therefore ran the analyses again but with only the four common temporal characteristics. The *z*‐scores method now indicated significant differences in the level of integration across advertisement and aggressive calls for *D. ebraccatus* compared to both *H. versicolor* and *H. cinerea* (Table 4). The comparison between *H. versicolor* and *H. cinerea* was not statistically significant. Random skewers analysis found no significant commonalities in **P** (no similarity in the pattern of integration across the call repertoire) between *D. ebraccatus* and *H. cinerea* but some commonalities in **P** for the other two species comparisons (some similarities in the pattern of integration across the call repertoire; Table 4).

### 3.3 Robustness of **P** to social environment

Random skewers analysis gave evidence for a common matrix structure for comparisons between *H. versicolor* advertisement calls produced at each of the three intermale distances (Table 5). In other words, **P** was not affected by the level of competition (Tables S19–S21).

**Table 5:** Random skewers analysis comparing **P** for advertisement calls given at each of three intermale distances, reflecting three different intensities of male–male competition

Intermale distances compared	*r*	*p*
1.8 m–0.9 m	.98	<.001
1.8 m–0 m	.98	<.001
0.9 m–0 m	.97	<.001

*N *=* *222 individuals. The null hypothesis is that there is no common matrix structure between calls given at the two different distances.

Winners and losers of *H. versicolor* contests had similar **P** matrix structure (Tables S22–S27) and levels of integration across the call repertoire. Comparisons of integration suggested similar levels of integration between advertisement and aggressive calls in winners and losers (Table 6). The random skewers analyses corroborated this finding, giving evidence for a common matrix structure both for advertisement and aggressive calls considered separately, and for a joint matrix containing both advertisement‐ and aggressive‐call characteristics (Table 6).

**Table 6:** Comparison of the strength of integration and the structure of **P** between winners and losers of staged aggressive interactions

Call type	PLS comparison		Random skewers		*N* winner	*N* loser
	Comparison effect size	*p*	*r*	*p*		
Adv	—	—	.94	<.001	151	72
Agg	—	—	.96	<.001	87	91
Adv and Agg	0.96	.17	.88	<.001	72	38

Comparison of the strength of integration (PLS comparison) was performed only on **P** containing the full set of advertisement‐ and aggressive‐call characteristics. Comparison of the structure of **P** (random skewers) was also performed separately for **P** containing either advertisement‐ or aggressive‐call characteristics only. The null hypothesis for the PLS analysis is that there are similar levels of integration between winners and losers; the null hypothesis for random skewers analysis is that there is no common matrix structure between winners and losers.

## 4 DISCUSSION

Our analysis of the two main signal types of treefrogs as an integrated signaling phenotype revealed both commonalities and divergence in **P** matrix structure across the three species. There was evidence for significant integration between advertisement‐ and aggressive‐call characteristics in all three species, which was largely driven by covariation among frequency characteristics. However, *H. versicolor* showed relatively weaker integration of aggressive and advertisement calls perhaps pointing toward these call types acting as separate modules, and both direct comparisons of the level of integration and the random skewers analyses indicated some differences between the species. We also found that **P** was robust to variation in the social environment (intensity of competition) in *H. versicolor*. Although we recognize the limitations of the inferences that can be made with a sample of three species, our between‐species comparisons nevertheless provide novel insights into signal evolution that could encourage more researchers to gather relevant data. In particular, these findings have important implications for the understanding of the evolution of complex signal repertoires, the potential for multivariate sexual selection to influence signal evolution and the nature of plastic behavioral responses to changing environmental conditions, which we discuss below.

### 4.1 Integration and the evolution of complex signal repertoires

We found that the three species had largely similar **P** matrix structures when considering the subset of acoustic characteristics common to all species’ advertisement and aggressive calls, although greater differences were revealed when we only considered temporal characteristics because all species had a strong correlation between the dominant frequencies of the two call types. *D. ebraccatus* had tight integration across the temporal characteristics of the two call types, with particularly large correlations between call durations and rise times. A similar, but weaker, pattern was found in *H. cinerea*, whereas the advertisement and aggressive calls of *H. versicolor* were clearly modular, with only a weak positive correlation between the duty cycles of the two call types. The strength of integration of signal repertoire components estimates the extent to which different signal types evolve independently of one another, and it is therefore intriguing that *H. versicolor*, the species with the greatest difference between its advertisement and aggressive calls (Figure 1), also showed the strongest evidence that these call types are separate phenotypic modules. We hypothesize that species differences in integration across the signal repertoire are related to between‐species differences in the acoustic similarity of their advertisement and aggressive calls, although this hypothesis must remain tentative until data are collected from more species. There is substantial variation among anuran species in the acoustic similarity of their advertisement and aggressive calls (Wells, 2007). Why these differences between species arose in the first place remains to be explored, as there have been few investigations of the origins and evolution of complex signal repertoires (Alexander, 1962; Castellano, Tontini, Giacoma, Lattes, & Balletto, 2002; Owen, 2003).

Although there have been few investigations of the integration of signal repertoires, several studies have now investigated whether different populations or species have evolved differences in **P** for a single signal, usually a signal involved in mate attraction. **P** is often relatively stable across populations within a species (Pitchers et al., 2013; Roff, Mousseau, & Howard, 1999; but see Hine, Chenoweth, Rundle, & Blows, 2009), but generally differs between species (Blankers et al., 2017; Roff et al., 1999). However, some cross‐species studies find evidence for a common structure of at least some matrix components, in particular the major axis of variation (Bertram et al., 2012). Our examination of the whole signal repertoire in three treefrog species revealed evidence for divergence in **P**, at least for temporal characteristics; in other words, we found evidence that species vary in the extent to which characteristics of a given signal covary with those of other signal types. We did not test whether divergence in **P** between species was most likely caused by selection or drift. Drift explained between‐population variation in **P** in three different cricket species (Pascoal, Mendrok, Wilson, Hunt, & Bailey, 2017; Roff et al., 1999), while a comparison of species with more divergent signal types found evidence for sexual selection generating variation in **P** between groups of species with discrete signal differences (Blankers et al., 2017). More studies across a broader taxonomic range of population and species variation in integration within and across signal types are badly needed to uncover the factors resulting in divergence or conservation in **P**.

The reason that phenotypic integration is such an important consideration for understanding the evolution of diversity in animal communication is that traits that covary may not evolve independently. Indeed, the response to selection on an integrated phenotype depends on the extent to which the multivariate selection surface aligns with axes of high genetic variation in the phenotype under selection (Cheverud, 1984; Lande & Arnold, 1983; Walsh & Blows, 2009). In the case of different signal types, these are given in different (albeit somewhat overlapping) contexts, and so two selection gradients (or one more general gradient) must be considered corresponding to selection acting on signals in each context. Measures of multivariate selection on mating signals have now been performed in many species (Blows et al., 2003; Brooks et al., 2005; Gerhardt & Brooks, 2009) and the estimated selection surfaces indicate complex selection acting on multiple signal traits. Aggressive signals have received much less attention in selection studies, and even univariate selection on variation in aggressive signals is rarely quantified (Tibbetts, Forrest, Vernier, Jinn, & Madagame, 2015). Nonetheless, a multivariate selection surface could conceivably be estimated for aggressive signals as well. If, however, mating and aggressive signals combine as a single integrated phenotype, then positive selection acting on the characteristics of one signal type may not result in a response to selection if this is countered by negative selection acting on characteristics of the other signal type (Moore & Moore, 1999).

The relationship between signal integration and the geometry of multivariate selection has consequences for not only the evolution of the signals themselves but also for the evolution of the animals producing them. For instance, communication signals related to mate recognition are expected to be under selection to diverge between closely‐related sympatric species (Hoskin, Higgie, McDonald, & Moritz, 2005; Nosil, Crespi, Gries, & Gries, 2007; Saetre et al., 1997), while in the same species, communication signals related to alarm signaling or agonistic resource defense may in fact be selected to converge (or to have not diverged) in structure (Drury, Okamoto, Anderson, & Grether, 2015; Tobias & Seddon, 2009; Wheatcroft & Price, 2015). Thus, if signal repertoires are tightly integrated, perhaps because of shared production mechanisms, yet selection pressures are divergent, then species diversification may be impeded. Eventually under continual divergent selection, either stronger selection on one signal type will drive the correlated evolution of others, or the integration between signal types will be eroded to the extent possible, allowing for diversification (Melo & Marroig, 2014; Sinervo & Svensson, 2002). The key point, however, is that diversification would likely take place more rapidly if the signal types were not integrated and could evolve fully independently in the first place.

### 4.2 Integration across contexts

We also examined how **P** was affected by changes in the social context of signaling and found that in *H. versicolor,* **P** was relatively unaffected by the level of social competition (both in terms of intermale spacing and relative competitiveness during a contest). This is of course only one axis of environmental variation, and there are many other potentially relevant environmental variables that may affect signal expression and **P**. Nevertheless, individual call characteristics are highly plastic with respect to the level of social competition in *H. versicolor* (Reichert & Gerhardt, 2012, 2013b; Schwartz et al., 2002; Wells & Taigen, 1986), so it is noteworthy that despite this plasticity, the overall pattern of interrelationships between call characteristics remained stable. If **P** had been heavily context‐dependent, this would have weakened our confidence in the between‐species comparisons of integration because male signals were recorded in different circumstances for each species. Furthermore, if **P** is stable, then multivariate selection on signal traits may have similar effects on **P** across contexts. Several other studies have found that **P** is robust to environmental conditions, although most of these studies examine effects of the physical, rather than social, environment. For instance, in great tits, *Parus major*, there were stable correlation structures between three plumage coloration traits across seasons and years (Hegyi et al., 2015), and in black field crickets, *Teleogryllus commodus*,** P** was stable when different populations were reared in a common garden environment, and when crickets were exposed to two different quality diets (Pitchers et al., 2013). **P** measured for morphological characteristics has also often been shown to be stable across different levels of environmental perturbation (Bossdorf & Pigliucci, 2009; Pigliucci & Kolodynska, 2002). In contrast, European robins, *Erithacus rubecula*, adjusted characteristics of their songs when exposed to anthropogenic noise such that songs given in noisy conditions had a more tightly integrated **P** than songs given in the absence of anthropogenic noise (Montague, Danek‐Gontard, & Kunc, 2013).

The evolutionary significance of a stable P across contexts depends on the context independence of selection itself, because multivariate selection on signals may also vary across environments (Cotton, Small, & Pomiankowski, 2006; Jennions & Petrie, 1997; Rodríguez, Rebar, & Fowler‐Finn, 2013). The alignment between **P** and the selection gradient determines the response to selection (assuming that **P** is a good estimate of **G**) (Cheverud, 1984; Lande & Arnold, 1983; Walsh & Blows, 2009), and therefore, when environmental conditions are variable, evolutionary studies must consider the context dependence of this alignment (Greenfield & Rodríguez, 2004; Ingleby, Hunt, & Hosken, 2010). Estimations of the context dependence of **P** and the selection gradient are particularly challenging in many cases of animal communication because selection acts on one individual, the signaler, and arises from the actions of another individual, the receiver, both of whom may show phenotypic plasticity in relevant behaviors. Relatively few studies have simultaneously estimated the context dependence of both signals and receiver response functions, but of those that have, several showed that the reaction norms for signals and preferences do not vary in parallel across contexts (Gerhardt & Mudry, 1980; Ritchie, Saarikettu, Livingstone, & Hoikkala, 2001; Rodríguez & Greenfield, 2003). While much work remains to be carried out, our methods to estimate **P** and its context dependence provide a useful tool toward understanding the context dependence of selection on animal signals.

## 5 CONCLUSIONS

The diversity and complexity of animal signals have continually fascinated and puzzled researchers in animal communication (Patricelli & Hebets, 2016). The development of new techniques to study signals as integrated phenotypes promises important new insights into the evolution of complex signal diversity. Our study used these methods to uncover potential links between signal type similarity and integration and demonstrate the robustness of integration to changing social conditions. Although our findings must remain tentative because of the small sample of study species, we describe an analytical framework that we encourage other researchers to apply to additional species to gain a greater understanding of the evolutionary significance of signal integration. There are many exciting topics for future investigation that would benefit both from studies of other anurans with more diverse communication systems and from other taxa, of which we will briefly mention two. First, our study species have relatively simple signals and signal repertoires, and it would be interesting to apply our approach to species with much more complex signals, and with many more signal types. Second, we only examined acoustic signals, but many species produce complex multimodal displays (Higham & Hebets, 2013). Comparisons of integration within and across signal elements in different modalities may be especially instructive. Are signal elements most integrated when produced in the same context, regardless of modality, or are signals in the same modality, even if produced in different contexts, more integrated with one another than they are with signals in a different modality produced in the same context? This has important consequences for the evolution of multimodal signals and also for hypotheses regarding the nature of the information (e.g., redundant or multiple messages) that can be most efficiently encoded by unimodal or multimodal signals (Hebets & Papaj, 2005).

## CONFLICT OF INTEREST

None declared.

## AUTHOR CONTRIBUTIONS

M.S.R. and G.H. designed the experiments and collected data, M.S.R. analyzed the data and wrote the paper, with editing by G.H.

## DATA ACCESSIBILITY

Data are available from the Dryad Digital Repository at https://doi.org/10.5061/dryad.d1k50.

## Appendix 1 Analyses removing derived variables

### 1.1

The analyses reported in the main text included some variables that were derived entirely from other variables in the analysis. First, duty cycle, which was measured for all call types of all species, is the ratio of call duration to call period. Second, the pulse rate of the aggressive calls in *H. cinerea* is the ratio of the pulse number to the call duration. Note that pulse rates reported for other species and call types are not entirely derived from other variables in the analysis. For both call types of *D. ebraccatus,* pulse rate is calculated as the number of pulses divided by the introductory note duration, but the call‐duration variable we used for this species was the full call duration which also includes supplementary click notes. We did not measure pulse rate for *H. versicolor* aggressive calls, and we did not include advertisement‐call duration as a variable for this species. Finally, only a portion of the advertisement call of *H. cinerea* contains pulses, so the pulse rate of this call type was not calculated from the full call duration. The derived nature of these variables may have affected analyses based on correlation matrix structure because these variables will be correlated by default. We argue that all of these variables are nonetheless of interest and include them in the analyses presented in the main text because they all have biological meaning and potentially different relationships with call characteristics across the signal repertoire. Nevertheless, to assess the sensitivity of our results to the inclusion of these variables, we repeated the analyses of integration and modularity with all species’ duty cycles and the aggressive‐call pulse rate of *H. cinerea* excluded.

#### Modularity and integration in advertisement and aggressive calls

Excluding duty cycle for all species and the aggressive‐call pulse rate for the most part did not change the qualitative interpretations of the results on the significance of modularity and integration across the signal repertoire in each species, but there were a few exceptions (compare Table 3 with Table A1). In particular, with duty cycle removed, *D. ebraccatus* no longer showed evidence for significant modularity of advertisement and aggressive calls for analyses only including temporal call characteristics. In contrast, for *H. cinerea*, analyses of temporal call characteristics in which the derived call characteristics were removed now showed evidence for significant modularity and no evidence for integration across the signal repertoire.

**Table A1:** Tests of modularity and integration of the signal repertoire, excluding derived variables

	Species	CR (CI)	*p*	*r*_PLS_	*p*	*N*
All characteristics	*D. ebraccatus*	1.07 (0.99–1.16)	.39	0.87	.001	28
	*H. cinerea*	1.07 (0.92–1.21)	.39	0.89	.001	28
	*H. versicolor*	0.78 (0.67–0.93)	.06	0.77	.001	111
Temporal characteristics only	*D. ebraccatus*	1.02 (0.96–1.11)	.12	0.87	.001	28
	*H. cinerea*	0.89 (0.74–1.23)	.03	0.6	.25	28
	*H. versicolor*	0.38 (0.30–0.59)	.004	0.34	.02	111

The CR statistic (with estimated 95% confidence interval) tests whether advertisement calls and aggressive calls are statistically separate modules. *r*_PLS_ tests whether there is significant integration across advertisement and aggressive calls. *N* indicates the number of individuals contributing mean values to the dataset.

#### Common structure of **P** across species

Removing duty cycle from the analyses of common characteristics generally strengthened the contrast between *D. ebraccatus* and the other two species (compare Table 4 to Table A2), adding support to our interpretation of the signal repertoire of *D. ebraccatus* being more integrated than that of the other two species. In contrast, the differences between *H. versicolor* and *H. cinerea* were weakened. We note that the analyses in which only temporal characteristics were considered, and in which duty cycle is excluded, now only contain three call characteristics per call type, and therefore may be less representative of the full range of signal variation.

**Table A2:** Comparison of the strength of integration and the structure of **P** across advertisement and aggressive calls for the characteristics common to all species and call types, with duty cycle excluded

Metric	Species	PLS comparison		Random skewers	
		Comparison effect size	*p*	*r*	*p*
All characteristics	*H.v.–D.e*.	0.98	.16	0.74	.03
	*H.v.–H.c*.	1.19	.12	0.86	<.001
	*D.e.–H.c*.	0.14	.44	0.66	.19
Temporal characteristics only	*H.v.–D.e*.	3.33	.0004	0.7	.24
	*H.v.–H.c*.	0.83	.2	0.83	.009
	*D.e.–H.c*.	3.39	.0003	0.63	.48

For each pairwise species comparison, we give the effect size for the comparison of *r*_PLS_ values and associated *p*‐value (Adams & Collyer, 2016), and the correlation and *p*‐value from random skewers analyses. Sample sizes for each species as in Table 3. The null hypothesis for the PLS analysis is that there are similar levels of integration between the two species; the null hypothesis for random skewers analysis is that there is no common matrix structure between the two species.

## Appendix 2 Subsampling of *H. versicolor* so that sample sizes were equal for all three species

### 2.1

The measurements of modularity and integration presented in the main text are based on a sample of 111 individual *H. versicolor* but only 28 individual *H. cinerea* and *D. ebraccatus*. The statistical power of our analyses of modularity and integration therefore differed between species. We note that the only explicit comparison of results between species is given in Table 4, in which we use the *z*‐scores method of Adams and Collyer (2016) that was designed to allow for comparisons of phenotypic integration independent of sample size. Thus, sample size should not be an issue for these analyses. Nevertheless, this is worth checking, and the reader will also naturally want to compare across species the results given in Table 3 on the integration and modularity of each species’ call repertoires. There are some apparent differences between species shown in this table that we allude to in the text although we do not explicitly compare species because of differences in the number of individuals measured and in the number and identity of the different call characteristics measured for each species.

To determine whether the large sample of *H. versicolor* influenced our estimates of integration and modularity, we used a resampling procedure to generate subsamples and then repeated the analyses reported in Tables 3 and 4. Specifically, we took 1,000 samples of 28 individuals from the H. versicolor dataset (each replicate was sampled without replacement) and for each sample, we estimated modularity using CR, integration using PLS, and compared integration levels to those of the other species using the compare.pls procedure and random skewers. Analyses were performed as in the main text.

#### Modularity and integration in advertisement and aggressive calls

Results for *H. versicolor* based on a resampling procedure with a reduced sample size were largely similar to results obtained from the full sample of individuals (compare Table 3 and Table A3). The primary difference was that the test of integration for the analysis with only temporal call characteristics was no longer statistically significant. This was not surprising, as Adams and Collyer (2016) point out that like most statistics, PLS analyses are affected by sample size.

**Table A3:** Tests of modularity and integration of the signal repertoire, with resampling procedure for *H. versicolor*

	Species	CR (CI)	*p* (CI)	*r*_PLS_ (CI)	*p* (CI)
All characteristics	*D. ebraccatus*	1.03 (0.94–1.12)	.24	0.86	.001
	*H. cinerea*	1.06 (0.94–1.16)	.43	0.88	.001
	*H. versicolor*	0.84 (0.83–0.85)	.055 (0.052–0.058)	0.776 (0.772–0.780)	.020 (0.016–0.025)
Temporal characteristics only	*D. ebraccatus*	0.97 (0.88–1.08)	.048	0.85	.001
	*H. cinerea*	0.95 (0.80–1.15)	.12	0.75	.03
	*H. versicolor*	0.59 (0.58–0.593)	.0067 (0.0060–0.0074)	0.523 (0.517–0.529)	.230 (0.217–0.244)

The CR statistic (with estimated 95% confidence interval) tests whether advertisement calls and aggressive calls are statistically separate modules. *r*_PLS_ tests whether there is significant integration across advertisement and aggressive calls. *N *=* *28 individual *D. ebraccatus* and *H. cinerea*, and each sample of *H. versicolor* also included 28 individuals sampled randomly from the original sample of 111 individuals. For *D. ebraccatus* and *H. cinerea*, results are identical to Table 3 because no resampling was performed. For *H. versicolor*, we present the mean value of all statistics obtained across 1,000 resampling replicates, along with the 95% confidence interval of this mean value.

#### Common structure of **P** across species

There were no qualitative differences in the results of the species comparisons when all call characteristics were included and we either used the full dataset for *H. versicolor* (Table 4) or the resampling procedure to give an identical sample size of all three species (Table A4). However, the effect sizes for the PLS comparison did change somewhat and were notably lower for the comparison between *H. versicolor* and *H. cinerea*. Effect sizes for the random skewers analysis were also somewhat lower. For the comparisons with only temporal call characteristics, the results were again largely qualitatively similar whether or not the entire set of *H. versicolor* individuals was used or when measures were calculated based on the resampling procedure. However, the random skewers comparison between *H. versicolor* and *D. ebraccatus* was statistically significant (*p *=* *.049) for the full *H. versicolor* dataset, but not significant for the resampled dataset (*p *=* *.12). In addition, the PLS comparison between *H. cinerea* and *H. versicolor*, which some would classify as marginally significant (*p *=* *.07) for the full *H. versicolor* dataset, was definitively not statistically significant for the resampled dataset (*p *=* *.25; and the effect size was much smaller). If anything, these findings bolster our conclusions that *D. ebraccatus* has a more integrated signal repertoire and a more divergent **P** than that of the other two species.

**Table A4:** Comparison of the strength of integration and the structure of **P** across advertisement and aggressive calls for the characteristics common to all species and call types, with resampling procedure for *H. versicolor*

Metric	Species	PLS comparison		Random skewers	
		Comparison effect size (CI)	*p* (CI)	*r* (CI)	*p* (CI)
All characteristics	*H.v.–D.e*.	0.83 (0.79–0.86)	.244 (0.234–0.253)	.681 (0.679–0.683)	.037 (0.034–0.040)
	*H.v.–H.c*.	0.61 (0.58–0.64)	.296 (0.288–0.304)	.788 (0.787–0.790)	<.001
	*D.e.–H.c*.	0.42	.34	.67	.08
Temporal characteristics only	*H.v.–D.e*.	2.27 (2.22–2.31)	.033 (0.030–0.037)	.671 (0.669–0.674)	.117 (0.110–0.124)
	*H.v.–H.c*.	0.77 (0.74–0.81)	.252 (0.244–0.261)	.789 (0.787–0.791)	.0020 (0.0017–0.0022)
	*D.e.–H.c*.	2.9	.002	.69	.101

For each pairwise species comparison, we give the effect size for the comparison of *r*_PLS_ values and associated *p*‐value (Adams & Collyer, 2016), and the correlation and *p*‐value from random skewers analyses. *N *=* *28 individual *D. ebraccatus* and *H. cinerea*, and each sample of *H. versicolor* also included 28 individuals sampled randomly from the original sample of 111 individuals. For the comparison between *D. ebraccatus* and *H. cinerea*, results are identical to Table 4 because no resampling was performed. For the other comparisons, we present the mean value of all statistics obtained across 1,000 resampling replicates, along with the 95% confidence interval of this mean value. The null hypothesis for the PLS analysis is that there are similar levels of integration between the two species; the null hypothesis for random skewers analysis is that there is no common matrix structure between the two species.
